# Modulating Gut Microbiota with Dietary Components: A Novel Strategy for Cancer–Depression Comorbidity Management

**DOI:** 10.3390/nu17091505

**Published:** 2025-04-29

**Authors:** Haochen Dai, Haiyi Yang, Rui Wang, Xuanpeng Wang, Xin Zhang

**Affiliations:** 1 Department of Food Science and Engineering, Ningbo University, Ningbo 315211, China; 2 Key Laboratory of Bio-Resource and Eco-Environment of Ministry of Education, College of Life Sciences, Sichuan University, Chengdu 610065, China; 3 SKL of Marine Food Processing & Safety Control, National Engineering Research Center of Seafood, Collaborative Innovation Center of Seafood Deep Processing, School of Food Science and Technology, Dalian Polytechnic University, Dalian 116034, China

**Keywords:** dietary patterns, diet-derived phytochemicals, gut microbiota, microbiota–gut–brain axis, prebiotics, probiotics

## Abstract

Background: Gut microbiota play a critical role in mediating the bidirectional association between cancer and depression. Emerging evidence indicates that adjusting the dietary component intake can significantly alter gut microbiota composition, thereby influencing the host’s metabolism and immune function. Changes in gut microbiota and their metabolites may represent key factors in preventing cancer–depression comorbidity. Methods: English publications were searched in databases including the Web of Science, Scopus, and PubMed using a series of keywords: “cancer”, “depression”, “gut microbiota”, “dietary components”, and related terms, individually or in combination. The search focused on preclinical and clinical studies describing the regulatory effects of dietary component interventions. Results: This narrative review summarizes the associations among gut microbiota, cancer, and depression, and synthesizes current evidence on the modulatory effects and mechanisms of specific dietary component interventions, including dietary patterns, probiotics, prebiotics, and diet-derived phytochemicals, on gut microbiota. On the one hand, these interventions inhibit abnormal proliferation signals in the tumor microenvironment and enhance anticancer immune responses; on the other hand, they modulate neurotransmitter homeostasis, suppress neuroinflammation, and improve mood behaviors through the gut–brain axis interactions mediated by microbial metabolites. Conclusions: The complex associations among cancer, depression, and gut microbiota require further clarification. Modulating gut microbiota composition through dietary components represents a novel therapeutic strategy for improving cancer–depression comorbidity. Regulated gut microbiota enhance immune homeostasis and intestinal barrier function, while their metabolites bidirectionally modulate one another via systemic circulation and the gut–brain axis, thereby improving both the tumor microenvironment and depressive-like behaviors in cancer patients while reducing the adverse effects of cancer.

## 1. Introduction

The human gut contains trillions of microbes that form a living community called the gut microbiota [1]. This complex microbial ecosystem plays a key role in maintaining human health and disease development [2]. From birth, gut microbiota play a crucial role in the innate and acquired immune response, regulating inflammation, infection, and assisting in the maintenance of a stable immune system [3].

Gut microbiota can metabolize some of the dietary fiber and phytochemicals in food that are indigestible by the gastrointestinal tract by secreting specific enzymes while producing metabolites such as short-chain fatty acids (SCFAs) and other active molecules [4]. These products not only provide energy to the host but also maintain intestinal barrier function and immune homeostasis by regulating tight junction proteins and immune cell activity [5]. The gut microbiota are dynamically influenced by antibiotic exposure, host aging, and dietary modifications [6]. Targeted dietary component modulation can enrich functional microbial communities, offering novel therapeutic targets for disease intervention.

The pathogenesis and treatment of cancer is one of the greatest challenges facing human society. Despite the extraordinary advances in our understanding of the mechanisms that cause cancer over the past 50 years, mortality rates remain high [7]. The etiology of cancer is complex and is associated with genetic factors, unhealthy lifestyles, environmental factors, and psychosocial factors, including depression and anxiety disorders. It has long been recognized that depression increases the risk of cancer [8]. Since the 1980s, numerous investigations have documented relationships between depression and immune function, endocrine regulation, cancer metastasis, treatment tolerance, and other biological processes [9]. Accumulating evidence from prospective epidemiological research indicates that depression acts as a risk factor for cancer development. Specific studies have demonstrated etiological connections [10,11], while other studies have not found a correlation [12]. From this, we can see that there is still controversy and uncertainty about depression as a cause of cancer. In addition to this, historical studies have found that depression is the most common comorbidity in most cancer types, affecting up to 20% of cancer patients [13].

Notably, beyond the controversial role of depression as a potential risk factor for cancer development, a growing body of evidence has firmly established that depressive symptoms are independently associated with poorer survival outcomes among cancer patients. For example, studies in patients with breast, colorectal, gynecological, lung, and prostate cancers have shown that severe depression is linked to worsened survival, with adjusted hazard ratios ranging from 1.36 to 1.76 and a pooled hazard ratio of 1.41 across cancer types [14]. Similar findings were observed in glioma patients, where depression was associated with a 42% increased risk of death in those with high-grade tumors and a significant reduction in survival duration [15]. Intriguingly, these associations remain robust even after accounting for anxiety, with depression demonstrating a persistent negative impact on survival in men and an amplified effect in women, whereas anxiety’s influence diminishes or reverses direction [16].

The mechanisms underlying these associations are multifactorial. Combinations of psychological and physiological factors appear to play a role. From a psychological perspective, cancer patients with comorbid depression exhibit a significantly poorer quality of life and are less likely to adhere to cancer treatment regimens, which may act as a causal factor in the association between depressive symptoms and high mortality in this population [17]. Additionally, depression induces chronic neuroinflammation and hypothalamic–pituitary–adrenal (HPA) axis dysfunction, both of which disrupt the systemic immune system [18]. Furthermore, gut microbiota dysregulation co-mediated by depression and cancer exacerbates neuroinflammation and depressive behaviors through the microbiota–gut–brain (MGB) axis. These interconnected pathways collectively contribute to the observed association between depressive symptoms and poor cancer survival.

Gut microbiota dysbiosis refers to the loss of key taxa, loss of diversity, changes in metabolic capacity, or proliferation of pathogens in the gut microbiota [19]. Patients with depression and cancer alike often experience symptoms of gut microbiota dysbiosis, with a positive correlation between the degree of dysbiosis and the severity of the disease [20]. Emerging evidence suggests that gut microbiota dysbiosis is not only associated with oncogenic effects and interference with the metabolism of cancer chemotherapy but also serves as a potential biomarker of cancer-treatment-related toxicity [21,22]. The disruption of the host microbiota leads to psychoneurological symptoms (PNS), the most typical of which are fatigue, anxiety, depression, and cognitive dysfunction [22]. These persistent and severe psychiatric symptoms may lead to delays in cancer treatment, a decreased tumor response, and a reduced quality of life for patients [23]. Notably, in recent years, it has also been shown that the gut microbiota can play a role in preventing or treating cancer, and may also concurrently improve PNS in cancer patients via the MGB axis [24,25]. This highlights the potential for therapeutic approaches that target the gut microbiota.

This narrative review summarizes the relationship between gut microbiota, cancer, and depression and then discusses the impact of various dietary components on the comorbidity of cancer and depression through the modulation of gut microbiota, highlighting the underlying mechanisms. This mechanistic framework may provide a scientific basis for the development of microbiota-directed nutritional intervention strategies.

## 2. The Interactions Between Depression and Gut Microbiota

### 2.1. Gut Microbiota in Depressed Patients

Depression, as a prevalent mental disorder worldwide, is characterized by core clinical features, such as persistent depressed mood and diminished interest in affective symptoms, accompanied by a marked reduction in multiple dimensions of the quality of life [26]. The differences between the gut microbiota of depressed patients and those of the healthy population have almost become a consensus among scholars in the relevant fields. One study compared the gut microecology of patients with first-episode depression with that of healthy volunteers and found significant differences between the two, as evidenced by an increase in the proportion of pathogenic bacteria and *Alistipes* and a significant decrease in the proportion of *Lachnospiraceae* and other beneficial bacteria in patients [27]. It has also been summarized that patients with major depression had a reduced abundance of *Prevotelaceae*, *Coprococcus,* and *Faecalibacterium* [28]. Taken together, at the phylum level, a high proportion of *Actinobacteriota* and a lower abundance of *Bacteroidetes* is a common feature of the changes in the bacterial microbiota of depression [29,30]. At the family level, in comparison with normal controls, depressed patients had a lower abundance of *Prevotellaceae* and a higher abundance of *Actinomycetaceae* and *Ruminococcaceae* in their intestinal tracts [29,31]. At the level of genera, patients with major depression had a lower abundance of *Sutterella* and *Faecalibacterium* in their intestinal tracts and a higher abundance of *Eggerthella* and *Collinsella* [32].

### 2.2. The Effect of Gut Microbiota on Depression

Recent studies have demonstrated that changes in gut microbiota can often influence the onset and progression of depression. Some studies have shown that mental stress may lead to gut ecological dysregulation and an imbalance of the gastrointestinal microbiota, and that ecological dysregulation may lead to low resilience and susceptibility to depression [33]. Physiological and psychological symptoms of depression change because of alterations in the structure of the gut microbiota, thus providing further evidence that gut microbiota may be involved in regulating the pathogenesis of depression.

The MGB axis is a bidirectional, regulatory axis between the gut microbiota and the brain. It encompasses the autonomic nervous system, enteric nervous system, central nervous system, HPA axis, central immune system modulation, and peripheral mechanisms, alongside other elements. Within this axis, the functions of each component interact with one another [34]. MGB axis dysfunction is an important part of the pathogenesis of depression, and the mechanisms involve various components of the MGB axis [35]. Depression can affect the composition and metabolic activity of the gut microbiota. Similarly, gut microbiota can affect the brain through humoral and neural mechanisms [36]. The dysregulation of certain specific strains of bacteria may lead to impaired social behavior, increased susceptibility to depression, and depressive disorders through the complex network of pathways of the MGB axis [37]. The reversal of gut microbiota effects occurs through a variety of pathways, including changes in neurotransmitters, changes in inflammatory factors, and changes in HPA axis functions.

Neurotransmitters are vital in governing various gastrointestinal functions and the gastrointestinal neuroendocrine and immune systems, maintaining dynamic homeostasis throughout the body and modulating the development and plasticity of the neural circuits associated with mood disorders such as depression [38]. Gut microbiota influence neuropsychiatric disorders mainly by affecting the synthesis of 5-hydroxytryptamine (5-HT), dopamine, and γ-aminobutyric acid (GABA) synthesis, thereby affecting the neuropsychiatric disorders of the host [39]. The HPA axis provides the primary biological response to stressful stimuli, and its overactivity serves as a contributing factor that causes depression [40]. The gut microbiota have an important association with the HPA axis, and altered HPA axis function can influence the compositional structure of the gut microbiota. Such compositional shifts may further induce microbial antigen expression, elevate host cytokine and prostaglandin levels, and trigger host HPA axis hyperactivity. This cascade can result in excessive cortisol production, thereby contributing to the onset of depression [41].

Through the MGB axis, gut microbiota exert a modulatory influence on depression and exhibit therapeutic promise. Metabolites derived from gut microbiota, such as SCFAs and neurotransmitters like 5-HT and GABA, exert modulatory effects on mood and behavior, thereby shaping the progression of depression.

## 3. Depression and Cancer

**Figure 1:**
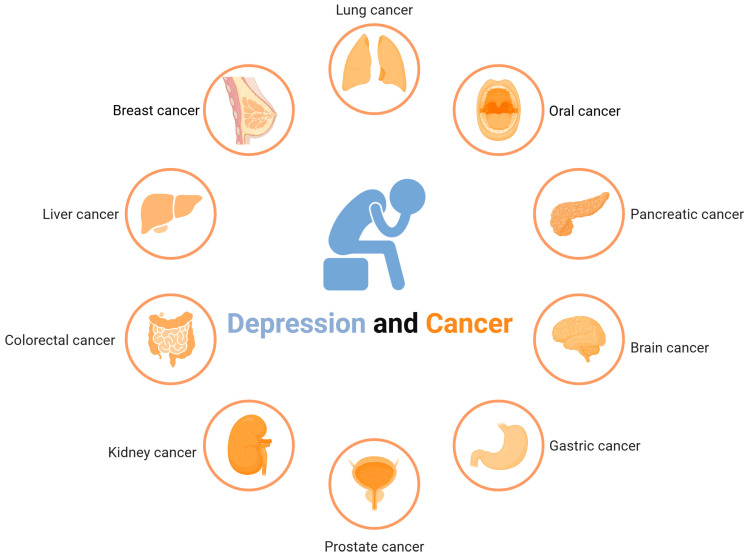
Multiple cancers are associated with depression [47,48,49,50,51,52].

Depression is a common comorbidity in cancer patients, and the prevalence of depression varies by cancer type (Figure 1). Depression is more likely to be associated with pancreatic, breast, lung, and oropharyngeal cancers [13]. Multiple factors, including inadequate pain management and polypharmacy, have been linked to an elevated likelihood of depression in cancer patients. Nevertheless, the etiological relationships between distinct cancer types and depression, as well as their underlying mechanisms, remain insufficiently elucidated. Malignant neoplasms pose a life-threatening risk, with cancer patients enduring physical distress arising from oncologic pain while simultaneously experiencing significant psychological strain. This dual burden often predisposes individuals to negative emotional states, such as anxiety and dysphoria [42]. Approximately 40% of patients with advanced malignancies, including carcinoma and hepatocellular carcinoma, encounter emotional distress at certain stages, impacting both their quality of life and their families [43]. Chemotherapy, hormone therapy, and targeted therapy represent the primary pharmacotherapeutic approaches for cancer treatment. Among these, an elevated incidence of depression has been observed in cancer patients undergoing chemotherapy [44]. Varying degrees of cognitive impairment have been associated with chemotherapeutic agents, including 5-fluorouracil, epothilone, cyclophosphamide, adriamycin, and paclitaxel [45]. Significantly, paclitaxel has been shown to substantially increase the incidence of depressive symptoms among cancer patients [46].

For decades, depression has been established as a common co-existing condition in cancer patients, rather than a predisposing factor for the disease. The cause-and-effect relationship between depressive symptoms and the cancer risk has undergone extensive investigation in multiple observational research studies. However, these findings have remained disputed, with substantial divergence evident in the analytical outcomes of these inquiries. Specifically, while some researchers endorse a causal link between depression and cancer development, others contend that any such association is either minimal or non-existent. Findings suggest that depression and anxiety disorders are correlated with a notably elevated likelihood of cancer development, cancer-related mortality, and overall mortality among cancer patients [53]. A Mendelian randomization study demonstrated a causal effect between genetically predicted depression and the breast cancer risk [54]. The biological link between depression and pancreatic cancer has been discussed, and it has been suggested that depression may be a precursor to pancreatic cancer [51]. A data meta-analysis showed that depression and anxiety were not associated with an increased risk for most cancer outcomes, except lung cancer due to smoking [55]. The evidence supports a bidirectional relationship between depression and cancer: depression influences carcinogenesis/progression, while cancer itself may precipitate depressive symptoms [56]. The psychological stress and social factors are associated with cancer incidence and survival rates, implying that depression may be associated with cancer development [57].

The association between cancer and depression constitutes a multifaceted and intricate subject within medical science. This interplay encompasses both biological pathways and psychosocial determinants that interact to modulate health outcomes and the quality of life among clinical populations. Specifically, it involves bidirectional influences where biological mechanisms and psychosocial stressors collectively shape the well-being of individuals in oncological and psychiatric care settings. Several of these potential mechanisms may be biological and may also be related to behavioral changes in patients with depression and anxiety. Depression and anxiety may lead to the aberrant activation of the HPA axis and high levels of norepinephrine and cortisol in the patient’s body, which suppresses the immune response to the tumor [58]. Depression and anxiety have also been associated with markers of inflammation. This leads to an increased likelihood of tumorigenesis and a poorer prognosis [59]. In addition, patients with mental disorders have a reduced awareness of early cancer symptoms, resulting in lower disease detection rates [60]. Following a cancer diagnosis, patients often experience persistent psychological distress, while disease-related physiological symptoms may be further amplified by cancer-induced psychological stress. Finally, there is evidence that cancer patients with depression and anxiety disorders are less likely to undergo surgery and receive less radiation and chemotherapy [61]. The lack of attention to these confounders in many previous studies may have affected the results observed in the studies. Overall, research on post-cancer depression has been fruitful in recent years, and studies on the relationship between depression and its resulting risk of cancer may have been underestimated.

## 4. Interaction Between Cancer and Gut Microbiota

### 4.1. The Variation of Gut Microbiota in Cancer Patients

The gut microbiota have been called the “second human genome”. The main methods used to classify the composition of the gut microbiota are 16S ribosomal RNA amplicon sequencing and metagenomic sequencing [62]. The taxonomic makeup of gut microbiota, including specific microbial species, has emerged as a promising biomarker for numerous diseases due to the predictive precision and clinical feasibility of its measurement and intervention [63]. Distinct disparities in gut microbiota composition across multiple taxonomic hierarchies have been identified between cancer patients and healthy individuals (Table 1).

Despite promising results on the predictive role of the gut microbiota in cancer immunotherapy, fully consistent results have not yet been obtained, possibly due to the dynamic, complex, and susceptible nature of the gut microbiota. Across diverse cancer types, both shared and patient-specific immunotherapy biomarkers have been identified. This phenomenon is attributed to the partial connectivity between the gut and other distant organs via their microbiota, which are hypothesized to influence human health significantly. Consequently, these primary interorgan associations—such as the gut–liver axis, the MGB axis, and the gut–lung axis—are collectively designated as analogous systems [80,81,82].

With the increasing understanding of the role of microorganisms in the development of cancer, a large number of studies have been conducted to investigate specific microecological changes during the course of illness in patients with various types of cancer. The results have shown that changes in gut microbiota tend to vary among patients with different cancers. Characteristic alterations in the characteristic microbiota and metabolites finalized under multiple studies may serve as biomarkers for the early screening diagnosis of cancer and even for treatment efficacy [83]. Studies have already been conducted to predict tumors, such as those of colorectal cancer (CRC), hepatocellular carcinoma, cholangiocarcinoma, lung cancer, and so on, using this method. For example, for CRC, researchers have found that patients have higher levels of *Fusobacterium nucleatum* in their feces than healthy populations, that tumor tissues contain higher levels of *Fusobacterium nucleatum* than adjacent healthy tissues, and that the abundance of *Fusobacterium nucleatum* is strongly correlated with a poor prognosis [84]; *Bacteroides fragilis* is strongly associated with CRC and has a significantly higher incidence of the disease in colon cancer tissues than adjacent healthy tissues [85]. Escherichia coli, the most common biomarker for CRC, is detected in patients’ feces. In addition to colorectal microscopy, which can be used as the gold standard for CRC diagnosis, changes in these three strains can also be used as microbial markers for CRC.

### 4.2. The Influence of Gut Microbiota on Cancer Development

**Figure 2:**
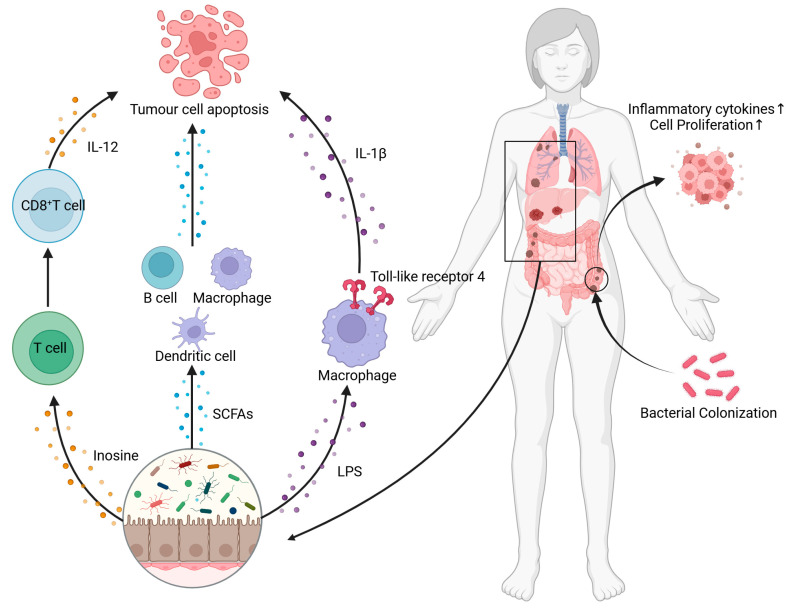
Gut microbiota affect the immune function and tumor microenvironment of the body in various ways [86,87,88]. Gut microbiota influence immune cells by secreting metabolites like Inosine and SCFAs, prompting immune cells to secrete cytokines that promote tumor cell apoptosis. Beneficial bacteria colonize the tumor microenvironment, killing tumor cells via mechanisms such as reshaping the tumor’s immune microenvironment and activating antitumor immune responses. Conversely, harmful bacteria colonizing it may cause tumor proliferation and even metastasis.

Gut microbiota can influence tumor occurrence, development, and prognosis by regulating the body’s immune balance and “tumor biological environment”. Gut microbiota can affect the body’s immunity and tumor microenvironment in various ways: (1) gut bacteria colonize the tumor and directly affect the tissue cells; (2) gut microbiota affect the tumor by regulating the local and systemic immune response of the tumor microenvironment; (3) gut microbiota affect the tumor locally and systemically through the secretion of metabolites or other protein substances absorbed by the human blood (Figure 2). The above three modes are not mutually exclusive, i.e., the same bacteria may affect tumors in different ways, and pathways involving the same signaling pathway may also include more than one mode of action.

The gut microbiota may contribute to cancer development and progression. Certain pathogenic bacteria can directly or indirectly cause genetic damage in the host or interfere with cellular replication [89,90]. In the presence of intestinal microecological disorders, pathogenic bacteria increase replication and release large amounts of toxins, inducing DNA breaks and genomic instability in the hosT-cell, which in turn facilitates tumorigenesis and progression [91]. Cytotoxin-associated protein A, produced by *Helicobacter pylori*, was the first bacterial protein shown to be involved in human cancer development. The colibactin and cytosolic lethal toxins produced by *Escherichia coli*, in contact with gastrointestinal epithelial cells, can cause DNA double-stranded damage and ultimately lead to tumor formation [92]. Inositol phosphate phosphatase D and cysteine protease-like virulence gene A, secreted by *Shigella flexneri*, and cytotoxin-associated gene A, secreted by *Helicobacter pylori*, induce the degradation of hosT-cell tumor suppressor gene p53, thus promoting the occurrence and development of cancer [93]. *Fusobacterium nucleatum*-derived FadA adhesins and *Bacteroides fragilis*-secreted metalloproteinase toxins mediate direct or indirect interactions with hosT-cellular components. *Helicobacter pylori* and *Bacteroides fragilis* target host epithelial cadherins through physical binding or paracrine modulation, inducing intercellular junction disruption and β-cyclin signaling pathway activation, thereby promoting cancer cell proliferation [94]. Both *Helicobacter pylori* and *Bacteroides fragilis* can activate host spermine oxidase, which generates hydrogen peroxide and reactive oxygen species (ROS), which is a key factor in cancer development. Both *Helicobacter pylori* and *Bacteroides fragilis* are capable of activating spermine oxidase in the host, which leads to the production of hydrogen peroxide and reactive ROS, and *Enterococcus faecalis* can produce extracellular superoxide, etc., which can be diffused into hosT-cells, and the resulting oxidized environment increases the possibility of DNA mutation in hosT-cells, causing DNA damage accumulation [95].

In contrast to pathogenic mechanisms, gut microbiota primarily suppress tumorigenesis through immune response modulation or microbial metabolite production, with certain species exerting antitumor effects via the activation of the tumor immune microenvironment [96]. Bacterial surface components like flagellin and lipopolysaccharides, acting as pathogen-associated molecular patterns, bind to Toll-like receptors on the surfaces of intestinal epithelial cells and dendritic cells (DCs), thereby inducing T-cell differentiation [97]. The body releases cytokines; generates inflammatory responses and activates the immune system; and influences the cellular responses of Th1/Tc1 to play a role in antitumor immune surveillance. Nontoxigenic *Bacteroides fragilis*, *Burkholderia cepacia*, and *Lactobacillus* stimulate DC maturation, enhance IL-12 production, and augment antitumor immunity [98]. *Enterococcus hiare* and *Barnesiella intestinihominis* were able to assist in combating tumors by increasing the infiltration of CD8^+^T-cells in the tumor microenvironment. Comparatively, *Salmonella* augments T-cell infiltration by down-regulating the expression of programmed cell death ligand 1 (PD-L1) on the surface of tumor cells [99]. In studies of prostate cancer, *Escherichia coli* could target and colonize prostate cancer foci to increase tumor-infiltrating immune cells, such as CD8^+^T-cells, Th17, DCs, macrophages, and natural killer cells, and to decrease the concentration of Tregs and vascular endothelial growth factor and thus to activate tumor immunity [100]. SCFAs are a series of fatty acids produced by gut microbiota through the fermentation of food substrates, including acetic acid, propionic acid, butyric acid, valeric acid, isobutyric acid, etc., among which, butyric acid is widely believed to inhibit colon inflammation and carcinogenesis [101]. Butyrate can act as a histone deacetylase inhibitor and up-regulate histone H3 acetylation levels and the expression of target genes, such as Fas, P21, and P27, and inhibit the growth of CRC and lymphoma [102].

Previous studies have found that the gut microbiota are associated with cancer-treatment-related PNS, including fatigue, anxiety, depression, sleep disturbances, cognitive deficits, and chemotherapy-induced peripheral neuropathy [103]. Elevated relative abundances of *Mycobacterium* have been positively correlated with an increased fear of cancer recurrence, while higher proportions of *Lachnospiraceae* and *Ruminococcus* taxa show an inverse relationship with this anxiety phenotype [104]. Variations in fatigue intensity have been linked to the prevalence of *Faecalibacterium* and *Prevotella*, whereas alterations in anxiety levels demonstrate a correlation with *Coprococcus* abundance [105].

**Figure 3:**
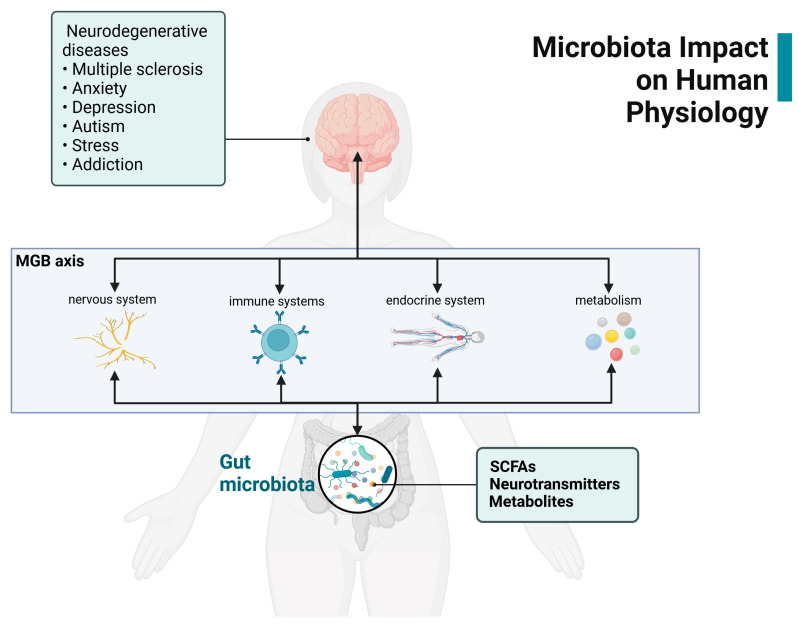
The microbiota–gut–brain axis links gut microbiota and multiple psychoneurological symptoms [25,107,108]. Gut microbiota produce SCFAs, neurotransmitters, and metabolites, interacting via the MGB axis with the nervous, immune, and endocrine systems, as well as with metabolism. These interactions influence neurodegenerative diseases and psychological conditions, such as multiple sclerosis, anxiety, depression, autism, stress, and addiction.

Understanding the pathways of the MGB axis could help identify innovative therapies for the treatment of cancer-associated PNS and could improve treatment-related outcomes in cancer (Figure 3). Recent studies have shown promise for interventions using prebiotics, probiotics, dietary intervention, or diet-derived phytochemicals [106].

## 5. Regulating Dietary Components Is a Promising Therapeutic Direction

**Figure 4:**
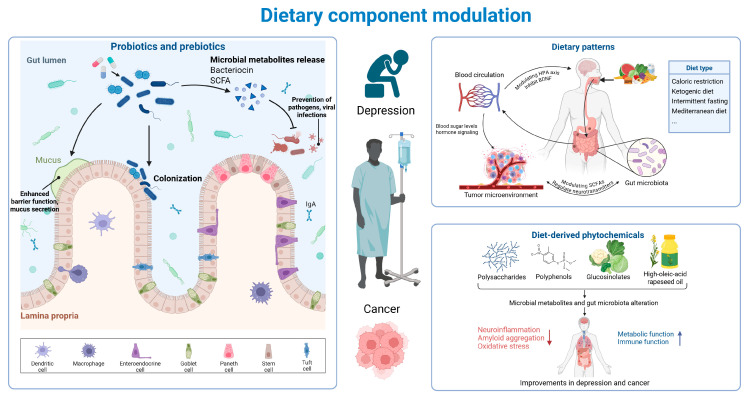
Dietary component modulation may be promising to improve cancer–depression comorbidity [109,110,111,112]. Probiotics and prebiotics release microbial metabolites, like bacteriocin and SCFAs, preventing pathogen and viral infections while enhancing barrier function and mucus secretion. Dietary patterns affect the body via blood circulation and modulate the HPA axis and the BDNF, and the gut microbiota influence the tumor microenvironment. Diet-derived phytochemicals alter microbial metabolites and gut microbiota and improve depression and cancer by reducing neuroinflammation and oxidative stress while enhancing metabolic and immune functions.

Cancer and depression are intricately linked, and dietary component interventions significantly contribute to regulating gut microbiota and ameliorating cancer and depression. The gut microbiota are significantly involved in both the pathogenesis and the management of cancer and depression. The modulation of gut microbiota through modifications in dietary components, such as dietary pattern adjustments, probiotics, prebiotics, and diet-derived phytochemicals to ameliorate cancer and depression is a mild and promising therapeutic strategy (Figure 4).

### 5.1. Dietary Patterns

Diet is an important source of nutrition with a substantial influence on human health and the trajectory of disease progression. Dietary interventions have evolved into viable complementary therapeutic approaches for a range of medical conditions, including cancer and depression [113]. Different dietary intervention modalities exert distinct effects on cancer development, where the link may be rooted in multiple biological mechanisms, with the regulation of gut microbiota assuming a central role [114]. Simultaneously, there is increasing scientific evidence of a relationship between dietary patterns and depression, supported by findings from animal experiments and population-based studies [115]. This dual regulatory effect highlights the potential of dietary interventions in the treatment of comorbid cancer and depression.

The main dietary patterns currently considered to have anticancer effects include caloric restriction, the ketogenic diet, and intermittent fasting. In addition, effects have been shown in preclinical trials through macronutrient-specific ratio diets, micronutrient supplementation or restriction, and following specific dietary patterns, such as the Mediterranean diet and plant-based diets. These approaches aim to inhibit tumor growth and improve cancer therapeutic outcomes by modulating systemic metabolic and immune responses [116]. Research has revealed that caloric restriction prevents tumor formation by enriching *Bifidobacterium* in the gut microbiota of mice. *Bifidobacterium* mediates the antitumor effect of caloric restriction through producing acetate, which promotes the accumulation of interferon-γ^+^ and CD8^+^ T-cells in the tumor microenvironment. This indicates that caloric restriction inhibits tumor growth by modulating gut microbiota composition [117]. Moreover, studies have demonstrated that using short-term starvation synergistically with a PD-1 blockade can successfully inhibit lung cancer progression and metastasis by decreasing the circulating levels of insulin-like growth factor 1 and down-regulating insulin-like growth factor 1 receptor signaling in tumor cells [118]. A ketogenic diet showed the inhibition of tumor progression and concomitant systemic inflammation in a mouse model of colon tumors without negatively affecting weight gain or muscle mass, which may help prevent cancer cachexia [119]. Protein-restricted diets may enhance the antitumor capacity of these key innate immune cells by altering the activity of tumor-associated macrophages. This diet reduces the infiltration of tumor-associated macrophages in the tumor, decreases tumor growth, and improves responses to immunotherapy [120]. A methionine-restricted diet contributes to reducing tumor growth and enhancing antitumor immunity by increasing the number and cytotoxic capabilities of CD8^+^ T-cells that infiltrate the tumor. Additionally, it counteracts tumor immune evasion by influencing the methylation of N6-methyladenosine and the translation of immune checkpoint molecules, such as PD-L1 and the V-domain Ig suppressor of T-cell activation, thereby inhibiting their expression in tumor cells [121]. In summary, dietary interventions show potential in cancer treatment by modulating metabolic pathways and enhancing immune responses. Certain dietary interventions can reduce blood glucose concentrations, modulate hormonal signaling, and influence the gut microbiota to directly inhibit tumor growth. Understanding these mechanisms can help develop precision nutritional strategies and provide new ideas for cancer treatment.

In cancer patients with commonly comorbid depression, dietary interventions can exert multifaceted effects through multiple pathways. Dietary interventions may influence the progression of depressive symptoms by modulating the HPA axis and brain-derived neurotrophic factor (BDNF) levels. Polyphenol-rich dietary patterns, such as the Mediterranean diet, may lower cortisol levels and reduce HPA axis disruption, whereas diets high in saturated fats and complex carbohydrates may inhibit BDNF and impair hippocampal neurogenesis [122]. The Mediterranean diet as an adjunctive treatment significantly improved the symptoms of moderately to severely depressed patients. In addition, the remission rate was four times higher in the dietary intervention group than in the control group, suggesting that the Mediterranean diet may offer an effective and feasible therapeutic strategy for the management of depression [123]. Calorie-restricted diets have been shown to significantly improve depressive symptoms in obese female patients [124]. Several studies have found that zinc-rich diets are negatively associated with depressive symptoms, suggesting that controlling depressive symptoms may be facilitated by modulating dietary zinc intake [125]. Flavonoid-rich diets may enhance BDNF levels, thereby improving depressive symptoms [126]. A ketogenic diet combined with regular voluntary exercise was implemented to reduce anxiety and depressive behaviors in mice [127]. In addition, diet is involved in oxidative stress, inflammatory responses, monoamine neurotransmitter regulation, and mitochondrial function, all of which are associated with the pathophysiology of depression. Therefore, rational dietary interventions may have preventive and therapeutic potential for depression.

As summarized above, dietary interventions have demonstrated substantial promise in modulating cancer and depression and their comorbidities. Research has indicated that certain dietary patterns, such as the Mediterranean diet and the ketogenic diet, can influence disease progression by modulating metabolic pathways, hormonal signaling, inflammatory responses, the HPA axis, and the balance of the gut microbiota. Gut microbiota, as a key interactive interface between diet and host, play a central role in these processes, influencing host metabolism and behavior, and providing new perspectives in the therapy of cancer and depression. Future studies need to further elucidate the complex interactions between diet, gut microbiota, and disease to develop more precise nutritional intervention strategies.

### 5.2. Probiotics and Prebiotics

Probiotics are defined as live microorganisms which, when administered in adequate amounts, confer a health benefit to the host [128]. Traditional probiotics are mainly derived from *Bifidobacterium*, *Lactobacillus*, and some other Lactic acid bacteria or Yeast, etc. [129]. As an exogenous intervention, probiotics have the effect of preventing diseases and enhancing immunity, and their anticancer ability and improvement of the side effects of cancer treatment are gradually being recognized and valued [130,131]. The core therapeutic mechanism lies in its regulatory effects on the gut microbiota. Probiotic supplementation demonstrates promising potential in managing comorbid cancer and depression. Research on cancer treatment through probiotics has been accumulated for many years, and its main mechanisms are to improve host fitness, enhance patients’ responsiveness to anticancer drugs, correct the gut microbiota dysbiosis induced by cancer treatments, reduce antibiotic use, and ameliorate the side effects accompanying cancer treatments [132]. For CRC, *Escherichia coli* Nissle, *Lactobacillus rhamnosus*, and *Lactobacillus plantarum* are known to enhance intestinal barrier integrity by increasing the expression of tight junction proteins, such as zona occludens-1, zona occludens-2, and Claudin-1. This mechanism alleviates the inflammatory microenvironment and facilitates mucosal healing [133,134]. Probiotics have been demonstrated to aid in enhancing the patient quality of life by inhibiting pathogenic invasions, lowering the risk of intestinal infections, and minimizing complications in CRC patients to impede tumor progression [135]. Eleven fecal bacterial strains were isolated from healthy humans. Their consortium induced IFN-γ^+^ CD8^+^ T-cells in mice, potentiated immune checkpoint inhibitor efficacy without colitis induction, and is currently undergoing clinical translation trials [136]. Gastric cancer patients receiving partial gastrectomy supplemented with *Lactobacillus plantarum* MH-301, *Bifidobacterium animalis* subsp. *Lactis* LPL-RH, *Lactobacillus rhamnosus* LGG-18, and *Lactobacillus acidophilus* exhibited enhanced nutritional and immune parameters [137]. A defined probiotic formulation markedly reduced radiation-induced mucositis severity/incidence and ameliorated systemic inflammatory biomarkers in cancer patients [138]. Probiotics not only alleviate various symptoms in cancer patients, but their regulation of gut microbiota may also influence neural activity and psychological states through the gut–brain axis, thereby exerting a positive impact on the overall therapeutic process. Probiotic supplementation with a mixture containing *Lactobacillus acidophilus*, *Lactobacillus casei*, and *Bifidobacterium bifidum* improved psychiatric symptoms while reducing insulin resistance and serum C-reactive protein levels in depressed patients [139]. A probiotic combination containing *Bifidobacterium longum* R0175, *Lactobacillus helveticus* R0052, or a placebo was given to healthy individuals for 30 days. The results showed that the probiotic-receiving group had significantly lower emotional distress than the control group [140]. Administering a probiotic combination containing *Bifidobacterium*, *Lactobacillus*, and *Lactobacillus casei* for 4 weeks reduced cognitive responses to sadness—specifically aggression and rumination—in healthy individuals without active mood disorders [137]. Overall, probiotics can show antidepressant effects in four ways, including the modulation of neurotransmitters, butyrate production, bacterial secreted proteins, and immunomodulation.

Prebiotics are defined as a substrate that is selectively utilized by host microorganisms conferring a health benefit [141]. According to FAO/WHO experts, prebiotics are generally not digested or absorbed by the human body but can be selectively utilized by human microorganisms, which confer a health benefit to the host by selectively stimulating microbiota that are considered important for maintaining the microecological balance [142]. In addition, they are considered alternatives to probiotics and can provide support for probiotics. Studies on cancer treatment through prebiotic intake have focused on animal experiments, and clinically it is mainly used as an adjunct to the rest of the therapeutic approaches, and its mild regulatory mechanism makes it a promising adjunct to cancer treatment. An in vivo study showed that jujube polysaccharide as a prebiotic had a protective effect against Azoxymethane/Dextran Sodium Sulfate-induced CRC in C57BL/6 mice, possibly preventing colon cancer by regulating the structure of the intestinal microbiota and alleviating colitis-induced intestinal malnutrition [143]. In a study targeting a mouse model of lung adenocarcinoma, Sintilimab combined with prebiotic treatment showed tumor growth inhibition and the modulation of immune cell homeostasis and may also be related to changes in the diversity of gut microbes [144]. The prebiotics inulin and mucin enhanced antitumor immunity and inhibited melanoma growth in C57BL/6 mice. The effect of inulin was even more pronounced, and it also enhanced the efficacy of mitogen-activated extracellular signal-regulated kinase inhibitors and delayed drug resistance, demonstrating the critical role of gut microbes in the fight against cancer [145]. The researchers found that the use of prebiotics attenuated liver and kidney damage induced by Sintilimab treatment in mice with lung adenocarcinoma and promoted the modulatory effect of Sintilimab on immune cells [144]. Prebiotic supplements are not as well researched for depression treatment as probiotics, but they are also an emerging gut microbiota-targeted therapy for alleviating behavioral disorders. Fructooligosaccharides/galactooligosaccharides reduce depression/anxiety in mice via corticosterone suppression, proinflammatory cytokine attenuation, hippocampal BDNF elevation, and microbiota modulation [146]. Twenty-six weeks of 200 mg daily resveratrol supplementation lowers depression scores and improves memory capacity in overweight elderly individuals [147].

In summary, accumulating evidence supports the therapeutic capacity of probiotics, prebiotics, and synbiotics to mitigate depression and cancer comorbidity by restoring gut microbial equilibrium. These interventions primarily act through microbial metabolites such as SCFAs, which regulate neuroimmune communication. Critical challenges persist, including individual variability in microbial responses, unresolved mechanistic links between specific microbial strains and host outcomes, and a lack of standardized protocols for clinical translation. Future research must prioritize elucidating strain-specific metabolic networks that bridge neurological and oncological pathways, alongside developing food-grade formulations of probiotics and synbiotics to enhance microbial resilience. Integrating multi-omics biomarkers with personalized dietary strategies will be essential to advance these interventions into scalable, evidence-based tools for managing comorbid conditions.

### 5.3. Diet-Derived Phytochemicals

Diet-derived phytochemicals represent a crucial category of dietary components that directly interact with both cancer pathophysiology and depressive disorders through gut microbiota-mediated pathways [148]. These phytochemicals exhibit anticancer properties by modulating microbial metabolites involved in carcinogen detoxification and immune checkpoint regulation [149]. Concurrently, their microbiota-dependent conversion produces neuroactive metabolites capable of crossing the blood–brain barrier to enhance hippocampal neurogenesis and 5-HT synthesis [150]. This dual-axis mechanism positions plant-derived compounds as unique dietary modulators targeting the shared MGB circuitry in cancer–depression comorbidities.

Recent studies have demonstrated that dietary phytochemicals intervene in cancer initiation and progression through multidimensional mechanisms involving the remodeling of gut microbiota composition and the regulation of microbial metabolic networks. For instance, catechins—a class of dietary polyphenols found in tea, cocoa, grapes, and apples—ameliorate microbial dysbiosis under conditions of depression or cancer [151,152]. Anthocyanins, abundant in berries, vegetables, and leaves, modulate gut microbiota by enhancing microbial diversity and the proportion of beneficial bacteria. Feeding black raspberries to a colitis-associated CRC mouse model was demonstrated to increase the relative abundance of beneficial bacteria in the fecal microbiota, accompanied by suppressed colitis and colonic tumorigenesis [153]. Dihydromyricetin, a natural flavanol, significantly alters gut microbiota composition and diversity [154]. In a colitis-associated CRC murine model, dihydromyricetin enhanced the chemotherapeutic efficacy of irinotecan by reducing the abundance of *Fusobacterium*. Another study revealed that dihydromyricetin enriched populations of *Bacteroides thetaiotaomicron*, *Bifidobacterium*, *F. prausnitzii*, and *Lactobacillus*, while decreasing the susceptibility to CRC carcinogenesis [155]. Neohesperidin, a citrus-derived flavanone glycoside, suppressed colorectal tumorigenesis in transgenic murine models through microbiota remodeling, specifically inducing the enrichment of *Firmicutes* and *Proteobacteria phyla* while reducing *Bacteroidota* abundance [156]. The ingestion of these compounds potently regulates gut microbiota composition. Metabolites generated by gut microbiota not only impact cancer therapy outcomes but also enhance patient mood through the gut–brain axis. Tea polyphenols in jasmine tea were found to modulate the gut microbiota of depressed rats, increasing the relative abundance of beneficial bacteria, such as *Mycobacterium anisopliae* and *Bradyrhizobium*, while decreasing *Ruminococcus* and *Butyrivibrio* [157]. Curcumin significantly alleviates anxiety–depression-like behavior and ameliorates intestinal dysbiosis and prefrontal metabolic disorders in mice with inflammatory bowel disease. This therapeutic effect is achieved through the modulation of gut microbiota and the MGB axis [158].

Plant polysaccharides are primarily sourced from cereals, legumes, tubers, fruits, vegetables, and algae. Due to their limited ability to traverse biological barriers and exert direct regulatory actions following oral administration, a broad hypothesis posits that their in vivo, indirect immunomodulatory and anticancer effects may rely on intermediaries such as gut microbiota and their metabolites [159]. For instance, ginseng polysaccharides combined with an anti-PD-1 monoclonal antibody slowed tumor progression in lung cancer model mice. This treatment also significantly increased the abundance of specific beneficial bacteria in the oral ginseng polysaccharide group [160]. Similarly, water-soluble polysaccharides from *Ganoderma lucidum* spores alleviated colitis, tumorigenesis, and gut microbiota dysbiosis. Water-soluble polysaccharide treatment enhanced gut microbiota diversity, reduced the relative abundance of *Lactobacillus reuteri* and *Bifidobacterium pseudolongum*, and increased *Bacteroides acidifaciens* and *Alistipes finegoldii* [161]. *Lycium barbarum* polysaccharides (LBPs) exhibit minimal intestinal absorption, with immunomodulatory effects arising primarily from gut microbiota interactions. LBPs elevate the relative abundance of *Lactobacillaceae*, *Bacteroidaceae*, and *Prevotellaceae* [162]. Polysaccharides also modulate concurrent depressive symptoms. For example, *Dendrobium officinale* polysaccharides alleviated anxiety–depressive-like behaviors in perimenopausal mice subjected to ovariectomy and chronic stress by restoring the gut microbiota balance, inhibiting prefrontal microglial activation, and normalizing HPA axis function [163]. Likewise, *Eucommiae cortex* polysaccharides mitigated anxiety–depressive-like behavior in chronic stress mice via the enrichment of *Lactobacillaceae*, suppression of microglial activation through multiple cellular pathways, and attenuation of hippocampal neuroinflammation [164].

In addition, the intake of high-oleic-acid cooking oils can modulate the gut microbiota. For example, high-oleic-acid rapeseed oil, virgin olive oil, high-oleic-acid peanut oil, etc. [165]. It has been shown that rapeseed oil can increase the relative abundance of *Akkermansia*, *Dubosiella*, and *Alistipes* [166]. *Akkermansia muciniphila* is involved in both CRC and the immune response, and increasing its abundance can help alleviate colitis-associated CRC [167]. In addition to this, a mixture of conventional rapeseed oil and high-oleic-acid rapeseed oil increased the relative abundance of *Parabacteroides*, *Prevotella*, *Turicibacter,* and *Enterobacteriaceae* and decreased the number of *Parabacteroides*, which is more beneficial to the health of cancer patients [168].

Glucosinolates, natural compounds found in cruciferous vegetables such as broccoli, cauliflower, cabbage, and kale, and their breakdown products, such as sulforaphane, exhibit an anticancer potential. Sulforaphane and its derivatives reduce the cancer risk by modulating the tumor microenvironment, inhibiting tumor stem cells, regulating microbiota, and exerting anti-inflammatory effects [169,170]. These compounds demonstrate efficacy against colon, breast, lung, bladder, and liver cancers [171], potentially through gut microbiota modulation, which attenuates chronic inflammation. Broccoli sprout supplementation in mice decreases *Proteobacteria* abundance while increasing *Firmicutes*, reflecting anti-inflammatory effects through microbiota remodeling [172]. Although glucosinolates are enzymatically cleaved to release isothiocyanates (ITCs), ITCs are further metabolized to cyanate in the liver by binding to glutathione via glutathione transferase, and may cause oxidative stress or mitochondrial dysfunction with certain side effects when ingested at high concentrations [173,174]. However, when consumed reasonably, they are usually harmless to healthy people and have some anticancer effects. Substituting butter with vegetable oils reduces the cancer-related mortality risk. Notably, double-low canola oil, characterized by a low erucic acid and a low glucosinolate content, demonstrated a 15% reduction in mortality risk, surpassing the 8% risk reduction observed with olive oil [175].

Allium species, including garlic, onions, and leeks, contain sulfides such as allicin, which show therapeutic potential against gastrointestinal cancers, hepatocellular carcinoma, leukemia, and skin cancers. These compounds inhibit pathogenic bacteria like *Helicobacter pylori*, a key driver of gastric cancer [176]. Such microbiota modulation may also alleviate depressive symptoms. Sulforaphane glucosinolate derivatives improved depressive-like behavior in chronically stressed mice by enhancing *Firmicutes* and *Actinobacteria* abundance, elevating brain 5-HT, dopamine, and BDNF levels [177]. Broccoli-derived sulforaphane glucosinolate ameliorated depressive-like behavior through *Lachnospiraceae*-mediated gut microbiota restructuring, tryptophan metabolism regulation, and systemic inflammation suppression, even in mice with hepatic ischemia-reperfusion injury [178].

In summary, modifying dietary patterns and intervening with probiotics, prebiotics, and diet-derived phytochemicals show potential in the management of cancer and depression comorbidity (Table 2). These components achieve dual disease interventions by remodeling the microbiota structure and modulating the host metabolic–immune–neuroendocrine axis: on the one hand, they inhibit aberrant proliferative signals in the tumor microenvironment and enhance the anticancer immune response, and on the other hand, they regulate neurotransmitter homeostasis, suppress neuroinflammation, and improve emotional behavior through microbial metabolite-mediated gut–brain axis interactions. The mechanisms of action include colony-dependent metabolite conversion, immune cell activity regulation, and HPA axis function regulation.

This study provides a scientific basis for formulating dietary component intervention strategies to improve cancer–depression comorbidity. Clinically, recommending dietary patterns such as the Mediterranean diet and ketogenic diet can effectively regulate gut microbiota composition, enhance intestinal barrier function, and reduce systemic inflammation. The administration of specific probiotic strains can optimize beneficial microbial communities, thereby inhibiting abnormalities in the tumor microenvironment and regulating neurotransmitter synthesis via the MGB axis to alleviate cancer–depression comorbidity. Prebiotic interventions can promote the production of SCFAs, enhance immune homeostasis, and improve both cancer-related inflammation and mood disorders. Additionally, diet-derived phytochemicals can regulate microbial metabolites to suppress neuroinflammation, enhance anticancer immune responses, and improve treatment tolerance. These dietary modulation strategies have the potential to serve as adjunctive therapeutic strategies for patients with cancer–depression comorbidity, offering a non-pharmacological approach to concurrently improve tumor control, mental health, and quality of life while minimizing treatment-related adverse effects. In the future, it is necessary to focus on the individualized characterization of microbiota–host interactions, to promote the transition of food ingredients from mechanistic research to clinical translation.

## 6. Conclusions

Complex interactions exist between cancer, depression, and gut microbiota. The targeted modulation of gut microbiota by altering food components, including dietary patterns, probiotics, prebiotics, and diet-derived phytochemicals, is a novel therapeutic strategy. Healthy gut microbiota improve immune homeostasis and intestinal barrier function, and the metabolites they produce are also bidirectionally regulated through the systemic circulation and the gut–brain axis, potentially improving both the tumor microenvironment and depressive-like behaviors in cancer patients and reducing the adverse effects of cancer. Future studies should aim to elucidate the mechanistic interactions between gut microbiota, depression, and cancer to develop more effective interventions to improve the outcome of patients with cancer–depression comorbidity.

## Figures and Tables

**Table 1:** The variation of gut microbiota in cancer patients.

Cancer Type	Microbes Enhanced in the Gut	Microbes Inhibited in the Gut	References
Gastric cancer	*Achromobacter*, *Citrobacter*, *Phyllobacter*, *Clostridium*, *Rhodococcus*, and *Lactobacillus.*	*Helicobacter*, *Blautia producta*, *Butyricoccus pullicaecorum*, and *R. faecis.*	[64,65]
Breast cancer	*Escherichia coli*, *Klebsiella sp_1_1_55*, *Prevotella amnii*, *Enterococcus gallinarum*, *Actinomyces sp. HPA0247*, *Shewanella putrefaciens*, and *Erwinia amylovora.*	*Eubacterium eligens*, *Lactobacillus vaginalis*, *Acinetobacter radioresistens*, and *Enterococcus gallinarum.*	[66]
Prostate cancer	*Bacteroides*, *Streptococcus*, *Rikenellaceae*, *Alistipes*, and *Lachnospira spp.*	*-*	[67,68]
Liver cancer	*Streptococcus*, *Klebsiella*, *Proteobacteria*, *Stenotrophomonas*, *Proteobacteria*, and *Veillonella.*	*Ruminococcus*, *Faecalibafcterium*, *Firmicutes*, *Ruminococcaceae*, *Butyricicoccus*, and *Lachnospiraceae.*	[69,70]
Lung cancer	*Bacteroides*, *Veillonella*, and *Fusobacterium.*	*Escherichia-Shigella*, *Kluyvera*, *Fecalibacterium*, *Enterobacter*, and *Dialister.*	[71]
Colorectal cancer	*Malassezia*, *Talaromyces*, *Trametes*, *Bacteroides fragilis*, *Akkermansia muciniphila*, *Clostridium hathewayi*, and *Alistipes finegoldii.*	*Pleosporaceae*, *Alemnaria*, *Blautia producta*, and *Roseburia faecis.*	[72,73,74]
Pancreatic cancer	*Proteobacteria*, *Synergistetes*, *Euryarchaeota*, *Bacteroides*, and *Verrucomicrobia.*	*Firmicutes*, *Actinobacteria*, and *Proteobacteria.*	[75]
Pancreatic cancer	*Streptococcus*, *Bifidobacterium*, *Subdoligranulum*, *Blautia*, *Romboutsia*, *Collinsella*, *Paeniclostridium*, *Dorea*, and *Atopobium.*	*Lachnospira*, *Bacteroides*, *Agathobacter*, *Fusobacterium*, *Parabacteroides*, *Paraprevotella*, *Butyricicoccus*, *Tyzzerella*, *Fusicatenibacter*, and *Sutterella.*	[65,76]
Esophageal cancer	*Bacteroidetes* and *Prevotella.*	*Faecalibacterium*, *Roseburia*, and *Blautia obeum.*	[77,78]
Cervical cancer	*Bacteroides* and *Parabacteroides.*	*Anaerostipes*, *Bifidobacterium*, *Blautia*, *Enterococcus faecalis*, *Dorea*, *Eubacterium*, *Ruminococcus*, and *Streptococci.*	[79]

**Table 2:** Impact of varied dietary components in cancer–depression comorbidity.

Types	Sample	Intervention	Key Findings and Conclusions	Reference
**Dietary patterns**				
KD	Humanized microbiome CRC mouse model; germ-free mice	KD consumption (dose not specified); microbiome transplantation	KD reduced colonic tumor burden via microbiome causality. KD-enriched stearic acid-producing bacteria suppressed tumor growth. KD antitumor effects mediated by microbiome-metabolite crosstalk.	[179]
MD-MIX, AOM, LFD	A/J male mice (AOM-treated; healthy LFD controls)	MD-MIX supplementation in LFD-fed mice; AOM injection (dose not specified)	MD-MIX reduced colonic lesions via apoptosis (LFD-MD-MIX). Counteracted CRC across diets via apoptosis-microbiome crosstalk.	[180]
CR, IF	Female mice with subcutaneous MC38 tumors	Six groups: Ad libitum, CR, IF, antibiotics+ad libitum, antibiotics+CR, antibiotics+IF.	CR suppresses MC38/4T1 tumors via gut microbiota-dependent mechanisms. *B. bifidum* restores CR antitumor effects via acetate-CD8^+^ T-cell activation. CR enriches *Bifidobacterium*; FMT replicates tumor suppression.	[181]
CR	CRC xenograft mice	CR: Initiated 12 days post-inoc. (100 mm³), 3 weeks duration.	CR suppresses isoleucyl-valine; elevates D-proline, phosphatidylcholine derivatives. *Lactobacillus*/*Parabacteroides*↑ correlate with antitumor metabolites (D-proline, 4-TMAB). CR reduces *Nitrospirae*/*Deferribacteres* linked to tumor-promoting pathways. Gut microbiota-metabolite axis mediates CR-driven CRC suppression.	[182]
**Probiotics and prebiotics**				
Probiotic combination (*Bifidobacterium longum*, *Lactobacillus lactis*, *Enterococcus faecium*)	Adults (18–70 years) with locally advanced nasopharyngeal carcinoma (n = 99)	Three capsules twice daily; 7 week	Severe OM reduced from 45.71% (placebo) to 15.52% (probiotics). Probiotics halved CCRT-induced T-cell depletion (CD4/CD8/CD3). Gut microbiota diversity restored to near-healthy levels. Immune resilience improved with probiotics (no major adverse effects).	[183]
*Bifidobacterium animalis*-containing probiotic yogurt	20 randomized metastatic renal cell carcinoma (mRCC) patients initiating VEGF-TKI therapy.	Two 4 oz servings of probiotic yogurt daily, continued for ≥12 weeks (stool sampling until week 12)	Probiotic supplementation achieved detectable *Bifidobacterium* enrichment in all treated patients. No significant difference in clinical benefit rates (70% vs. 80%) between groups. *Barnesiella intestinihominis* and *Akkermansia muciniphila* abundance strongly correlated with clinical benefit. First prospective RCT demonstrating probiotic-driven microbiome modulation in mRCC.	[184]
*Bifidobacterium longum*, *Lactobacillus acidophilus*, *Enterococcus faecalis*	159 breast cancer patients; Sprague–Dawley rats (hippocampal damage model)	Three capsules (0.84 g each) twice daily during chemotherapy (4–6 cycles, 21 day/cycle)	Probiotics reduced CRCI incidence by 32% and improved MoCA/SDS/SAS scores post-chemotherapy. Probiotics increased *Faecalibacterium*, reduced *Escherichia*, and modulated metabolites. p-Mentha-1,8-dien-7-ol reversed chemotherapy-induced hippocampal oxidative stress, synaptic injury, and glial activation. Probiotics prevent CRCI via gut microbiota-metabolite axis targeting p-Mentha-1,8-dien-7-ol.	[185]
*Lacticaseibacillus paracasei Shirota*, *Bifidobacterium breve* Yakult, galacto-oligosaccharides	73 esophageal cancer patients undergoing NAC	LBG+EN: 600 mL EN, 3 g probiotics, 15 mL GOS daily (pre-NAC to end)	Higher *Anaerostipes hadrus* and *B. pseudocatenulatum* linked to reduced FN/severe diarrhea (*p* < 0.05). Pre-NAC *A. hadrus* levels predicted FN risk (OR = 0.11) and post-NAC levels correlated with acetic/butyric acid. Gut microbiota profiling may identify LBG+EN responders, aiding NAC adverse event prevention strategies.	[186]
**Diet-derived phytochemicals**				
Stigmasterol	Balb/c mice bearing subcutaneous hepatocellular carcinoma	Oral administration of stigmasterol at doses of 0 (control), 50, 100, or 200 mg/kg every 2 days for 3 weeks	Activated apoptotic proteins (Caspase3, Bax, P53) and blocked Cyclin D1. Enriched beneficial gut microbiota (e.g., *Lactobacillus*). Lowered Treg/CD8^+^ ratios and boosted IFN-γ^+^ CD8^+^ T-cells.	[187]
Quercetin	BALB/c mice (BCRD model induced by 4T1 cells + CORT); primary hippocampal neurons (induced by LPS + CORT)	In vivo: quercetin treatment in BCRD mice. In vitro: hippocampal neurons treated with quercetin. PTGS2 overexpression to validate mechanism	Restored gut-lipid balance, suppressed PTGS2 in BCRD mice. PTGS2 binding inhibited ferroptosis, restored monoamines. Improved behavior/immunity, reversed by PTGS2 overexpression.	[188]
Defatted rice bran	AOM/DSS-induced colitis-associated CRC rat model	Defatted rice bran supplementation	DRB enriched beneficial bacteria, suppressed harmful taxa. Boosted SCFA production, restored mucus/gobleT-cell integrity. Prebiotic potential via microbiota modulation lowers CRC risk.	[189]
Garcinol	HFD-induced obese mice with AOM/DSS colitis-associated colon cancer	0.05% dietary garcinol supplementation	HFD exacerbated colon carcinogenesis; garcinol ameliorated progression. Garcinol modulated *Alistipes*/*Romboutsia* microbiota and oncogenic gene expression. Garcinol suppressed obesity-driven CRC via microbial/genomic regulation.	[190]

CCRT: concurrent chemoradiotherapy; OM: oral mucositis; KD: ketogenic diet; CRC: colorectal cancer; MD-MIX: Mediterranean diet mix; AOM: azoxymethane; LFD: low-fat diet; RCT: randomized controlled trial; CR: calorie restriction; IF: intermittent fasting; FMT: fecal microbiota transplantation; 4-TMAB: 4-trimethylammoniobutanoic acid; mRCC: metastatic renal cell carcinoma; VEGF-TKIs: vascular endothelial growth factor-tyrosine kinase inhibitors; CRCI: chemotherapy-related cognitive impairment; MoCA: Montreal cognitive assessment; SDS: self-rating depression scale; SAS: self-rating anxiety scale; GOS: galacto-oligosaccharides; NAC: neoadjuvant chemotherapy; FN: febrile neutropenia; OR: odds ratio; SCFA: short-chain fatty acids; HFD: high-fat diet.

## Data Availability

No data were used for the research described in the article.

## Abbreviations

The following abbreviations are used in this manuscript:

SCFAs	short-chain fatty acids
PNS	psychoneurological symptoms
MGB	microbiota–gut–brain
HPA	hypothalamic–pituitary–adrenal
5-HT	5-hydroxytryptamine
GABA	γ-aminobutyric acid
CRC	colorectal cancer
ROS	reactive oxygen species
DC	dendritic cells
PD-L1	programmed cell death ligand 1
BDNF	brain-derived neurotrophic factor
LBPs	Lycium barbarum polysaccharides
ITCs	isothiocyanates
